# P-696. Rapid estimates of RSV disease burden in the United States

**DOI:** 10.1093/ofid/ofaf695.909

**Published:** 2026-01-11

**Authors:** Emily Carter, Diba Khan, Li Deng, Imelda Trejo, Owen Devine, Koumans Emily, Michael Melgar, Heidi L Moline, Ismael Ortega-Sanchez, Christopher A Taylor, Gordana Derado, Fiona P Havers, Monica E P Patton

**Affiliations:** Centers for Disease Control and Prevention, Atlanta, Georgia; Centers for Disease Control and Prevention, Atlanta, Georgia; Centers for Disease Control and Prevention, Atlanta, Georgia; Former Centers for Disease Control and Prevention, Atlanta, Georgia; Centers for Disease Control and Prevention, Atlanta, Georgia; Centers for Disease Control and Prevention, Atlanta, Georgia; Centers for Disease Control and Prevention, Atlanta, Georgia; US-CDC, Atlanta, Georgia; Centers for Disease Control and Prevention, Atlanta, Georgia; Centers for Disease Control and Prevention, Atlanta, Georgia; CDC, Atlanta, Georgia; Centers for Disease Control and Prevention, Atlanta, Georgia; Centers for Disease Control and Prevention, Atlanta, Georgia

## Abstract

**Background:**

During the fall and winter in the United States, RSV is the leading cause of infant hospitalization and an important cause of morbidity and mortality among older adults and adults with certain chronic medical conditions. Real-time estimates of RSV burden during the respiratory virus season are needed to track seasonal trajectory, population impact, and health system burden.

**Figure 1:**
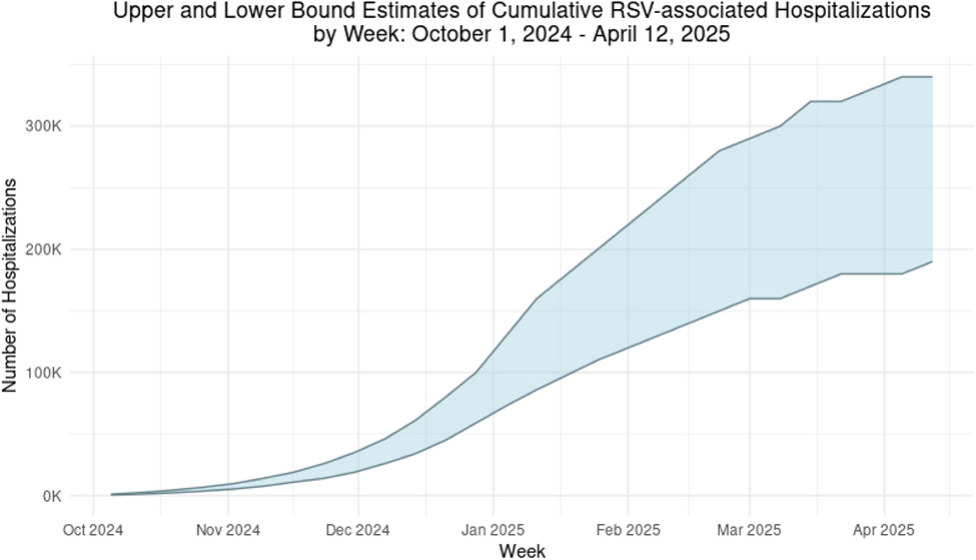

**Methods:**

For the October 2024-April 2025 respiratory season, the US CDC performed age-based statistical and mathematical modeling to generate near real-time, cumulative estimates of RSV-associated outpatient visits, hospitalizations, and deaths in the US. Estimates were publicly posted online each week. Weekly estimates of lab-confirmed RSV-associated hospitalizations from the RSV Hospitalization Surveillance Network (RSV-NET), adjusted for test sensitivity and likelihood of laboratory testing, were extrapolated to a national scale by applying an age-specific conditional autoregressive model with site-level random effects. Outpatient visits and deaths were estimated using an age-specific probabilistic multiplier model applied to the national RSV hospitalization estimates. The number of deaths per hospitalization was based on the observed mortality rates in RSV-NET, adjusted to include the proportion of RSV-associated deaths outside the hospital.

**Results:**

Applying the model to preliminary RSV-NET data, RSV contributed to approximately 4.7 million outpatient visits (range: 3.4–6.3 million), 250,000 hospitalizations (range: 180,000–340,000), and 15,000 deaths (range: 9,800–22,000) in the US (total population 330 million) between October 2024 and April 2025 (Fig 1). The highest outpatient and inpatient care rates were observed among children aged 0-23 months. In absolute numbers, the combined burden of outpatient visits and hospitalizations was similar among children < 5 years and adults ≥ 65 years. RSV-related mortality was highest among adults aged ≥ 65 years.

**Conclusion:**

Rapid estimation of RSV-associated disease burden is important for situational awareness of weekly changes in RSV incidence, seasonal epidemiology, and impact on the US health system and population. Rapid RSV burden estimates also help to inform the timing and effectiveness of prevention and immunization strategies.

**Disclosures:**

All Authors: No reported disclosures

